# The Study of Ice-Binding Protein Oligomeric Complexes

**DOI:** 10.3390/ijms262411790

**Published:** 2025-12-05

**Authors:** Galina A. Oleinik, Maria A. Kanarskaya, Na Li, Alexander A. Lomzov, Vladimir V. Koval, Svetlana V. Baranova

**Affiliations:** 1 Institute of Chemical Biology and Fundamental Medicine, Siberian Branch of the Russian Academy of Sciences, Novosibirsk 630090, Russiakoval@1bio.ru (V.V.K.); 2 Department of Natural Sciences, Novosibirsk State University, Novosibirsk 630090, Russia; 3 National Facility for Protein Science Shanghai, Shanghai Advanced Research Institute, Shanghai 201210, China

**Keywords:** ice-binding protein, oligomeric state, MALDI, native gel electrophoresis, isothermal titration calorimetry, atomic force microscopy, small-angle X-ray scattering, molecular dynamics simulation

## Abstract

Proteins play an important role in living organisms, and, for most of them, the function depends on their structure. There are some proteins that have similar properties but different structures. An example of this is ice-binding proteins (IBPs), which have different structures but share the ability to bind to ice. Many organisms have evolved such proteins to help them survive in cold environments. Therefore, it is important to study the oligomeric state of the active form in solutions. The activity of IBP is related to the area of their ice-binding site. We have demonstrated the presence of oligomeric forms of protein in solution using multiple techniques, such as mass spectrometry, native gel electrophoresis, atomic force microscopy (AFM), isothermal titration calorimetry (ITC) and small-angle X-ray scattering (SAXS). It is noteworthy that, to date, there have been no reports of the oligomerization of ice-binding protein from Longhorn sculpin. Additionally, our findings suggest that larger molecules may influence the ability of proteins to bind to ice. In our study, the ice-binding protein forms elongated assemblies with limited intermonomer interfaces. The combination of SAXS and AFM data indicates a structure that combines compactness and flexibility and probably consists of four monomeric units. The employment of molecular modelling methodologies resulted in the attainment of a tetrameric complex that is in alignment with AFM data. Details of oligomers observed using the methods in our study emphasize the importance of different techniques that complement each other in resolving structural features. Additionally, we suggest that the protein particles, which were dispersed on the surface, exhibit softness or the form planar complexes with loose quaternary structures. It is conceivable that, depending on ionic strength and/or temperature, the various oligomeric forms of the ice-binding protein form thermodynamically more favorable complexes than their monomeric forms.

## 1. Introduction

Proteins play an important role in living organisms, and for most of them, the function depends on their structure. For most proteins, a well-defined spatial structure is essential [1,2]. However, not all proteins can be studied experimentally to determine their function, and it is usually necessary to rely on sequence similarity to other proteins whose function is known. This allows us to predict the function of unknown proteins based on similarities in their sequences [1]. At the same time, there are some proteins that have similar properties but different structures. An example of this is ice-binding proteins (IBPs), which have different structures but share the ability to bind to ice surface.

Ice-binding proteins have been found in various cold-adapted organisms that can survive extremely low temperatures [3,4]. IBPs are highly diverse and differ in size, structure and function [4,5,6]. They exhibit a range of activities in different organisms, such as retaining liquid during freezing, promoting ice structuring and reducing freezing–melting temperature hysteresis [5,7,8]. The remarkable properties of IBPs have opened up prospects for their use in various areas of human activity. These proteins are valuable biomolecules in the food industry, where they improve the texture and preservation of frozen foods, such as in ice cream production [9,10,11]. In agriculture, IBPs enhance frost resistance [12,13]. They are also applied in cryosurgery [14] and cryopreservation of cell [15,16], tissues [16,17] and organs [18,19,20]. Additionally, IBPs have potential applications in aviation to prevent icing [21,22].

IBPs are relatively small (3–31 kDa) [23]. One way to increase the efficiency of their interaction with ice crystals is to increase the interaction area achieved by enlarging the protein molecule [24]. IBPs can assemble into their oligomeric forms, increasing their antifreeze activity [18,25]. Dimeric forms have been shown for some antifreeze proteins [24,26,27], and molecular modelling of oligomers has been performed [28]. Studies have shown that hybrid IBP constructs containing multiple ice-binding sites have enhanced activity compared to their single-domain counterparts [25,29]. Thus, recombinant dimers and high-order oligomers of Antarctic eel pout (*Lycodichthys dearborni*) antifreeze proteins have been used as examples to demonstrate an approximately twofold increase in antifreeze activity compared to the monomer [25,30]. The activity of this antifreeze is equivalent to the area of the ice-binding site [25]. While increasing the interaction area of a protein with ice can significantly increase its activity, this should be confirmed experimentally on a case-by-case basis [31]. Strong binding is more likely to result from the complementarity of the ice and protein surfaces. At the same time, the contribution of hydrogen bonds is probably secondary to van der Waals contacts [32].

Therefore, determining the oligomeric state of the catalytically active form of a protein in solution is extremely important for understanding the mechanism of action of an ice-binding protein in organism cells. For some proteins, such as IBP from longhorn sculpin (*Myoxocephalus octodecemspinosis*), establishing the structure and the presence of oligomeric forms is challenging [33]. As demonstrated previously, this protein has been shown to exhibit antifreeze activity [34] and influence the growth and shape of ice crystals [35]. Herein, we focused on the study of oligomeric states using multiple techniques, such as mass spectrometry, native gel electrophoresis, atomic force microscopy (AFM), isothermal calorimetry (ITC) and small-angle X-ray scattering (SAXS). Our findings demonstrate that protein exhibit distinct oligomeric forms in solution. It is noteworthy that, to date, the oligomerization of longhorn sculpin IBP has not been reported. It can be hypothesized that the presence of oligomers in the solution of IBPs enhances the binding affinity to the ice surface. It is crucial to ascertain the optimal structure to understand the interaction mechanisms between these proteins and ice.

## 2. Results and Discussion

The object of the study was an ice-binding protein from longhorn sculpin (*M. octodecemspinosus*) fish. At the high concentrations required for structural studies, protein tended to aggregate and precipitate [36]. We were interested in exploring the propensity for oligomerization of the protein from longhorn sculpin and what possible oligomeric structures, if any, were found.

### 2.1. Protein Obtaining and Purification

For structural studies, we used the protein obtained using the recombinant method. The isolation of IBP followed the procedures developed and described in ref. [37], with some modifications. The protein was extracted on a HisTrap HP column (Healthcare, Chicago, IL, USA), followed by further purification using gel filtration.

The protein was characterized via mass spectrometry on an Orbitrap Q Exactive HF high-resolution mass spectrometer (Thermo Scientific Inc., Waltham, MA, USA). MS/MS mass spectra of the peptides after trypsinolysis were obtained, which enabled the identification of the protein. Peptide identification was performed using Proteome Discoverer software (v 3.1, Thermo Fischer Scientific) and the search was conducted against a local database consisting of the protein sequence. The Sequest protein identification system, with a tolerance of 10 ppm, resulted in 99% protein coverage (Figure S1A). The protein preparation was electrophoretically homogenous (Figure S1B).

### 2.2. Structural Assessment of IBP Using Circular Dichroism

IBP studies are usually performed in solutions that mimic the natural physiological environment: pH from ∼6 to ∼8 with a minimum concentration of salts and sugars. Changes in pH and ionic strength can considerably affect protein activity [38]. Therefore, it was necessary to evaluate the effect of the composition of the protein in a solution on its conformation and stability. Additionally, the experimental conditions (buffer composition, pH and salt concentration) usually depend on the conditions of the structural analysis method. We analyzed the secondary structure of the protein in different buffer systems and compared it with literature data to exclude the influence of the environment.

**Figure 1:**
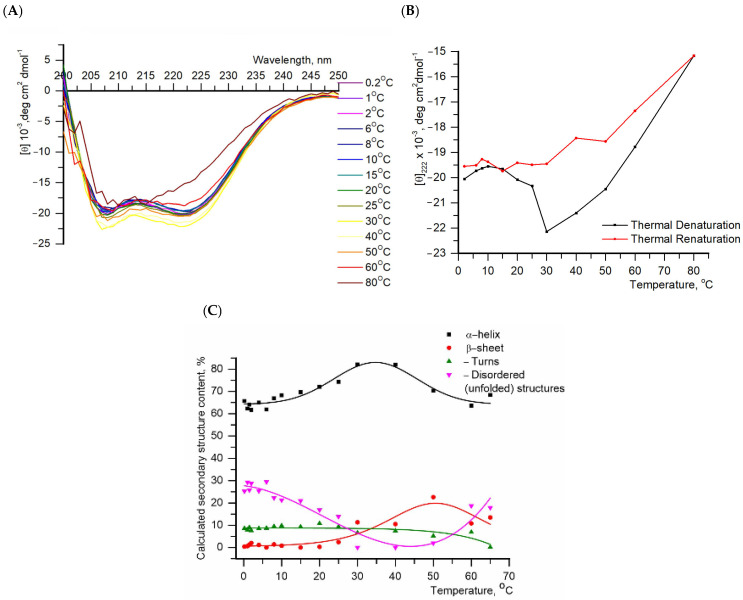
(**A**). Circular dichroism spectra of protein P80961 from *Myoxocephalus octodecemspinosus* at different temperatures. The protein concentration was 0.2 mg/mL, and the buffer was 20 mM HEPES pH 7.6, 100 mM NaCl; (**B**). Thermal denaturation (temperature ranges 0.2–80 °C) and renaturation (temperature ranges 80–6 °C) of IBP profiles at 222 nm, respectively; (**C**). The proportion of secondary protein structure as a function of temperature, obtained by detecting protein denaturation. Solid lines in (**B**,**C**) are envelope lines constructed for clearer data visualization.

We show that the secondary structure of protein in the temperature range from 0 °C to 35 °C in buffer containing 20 mM HEPES pH 7.6 and 100 mM NaCl was mainly represented by α-helixes and unstructured elements (Figure 1), which is in agreement with the results published elsewhere [34,35,36]. Circular dichroism data showed that the α-helix fraction for IBP was about 60%, which was consistent with the value calculated from the amino acid sequence using several secondary structure prediction algorithms (analyzed using resource: https://web.expasy.org/protscale/ (accessed on 1 December 2025)). Furthermore, the protein also had a structure predominantly composed of α-helices in phosphate buffer (pH 7.4) with 100 mM NaCl [37]. In this way, the structure of a protein is independent of the buffer systems in which it is contained.

The sample was first slowly cooled, then a temperature series of CD spectra was recorded in the range of 0.2–80 °C (Figure 1A). To study the effect of temperature on protein secondary structure. The temperature dependencies of the relative fraction of secondary structure types (α-helix, β-sheet, turn and disordered structure) are shown in Figure 1C.

The CD spectra (Figure 1A) of the recombinant IBP obtained here and in Deng et al. [35] for the wild-type protein isolated from longhorn sculpin (*M. octodecemspinosus*) serum coincided well. As a function of temperature (Figure 1B), the CD signal illustrated two transitions: the first from 15 °C to 40 °C and the second from 40 °C to 80 °C. At low temperatures (0–10 °C), the protein contains 65% α-helix (Figure 1C). The temperature increase up to 30 °C resulted in protein folding (an increase of up to 85% of the α-helix fraction, Figure 1C).

Denaturation of the protein starts at 40 °C. The denaturation and renaturation curves obtained at 222 nm did not coincide (Figure 1B). Only one transition was observed during the protein renaturation. Cooling of the protein did not lead to structure recovery due to irreversible unfolding. A similar effect was also noted by Deng et al. [35]. In study [35], the authors investigated a native protein whose sequence differs from that of our recombinant protein. However, molecular modeling of their structures revealed no significant structural differences between them (Protein molecular dynamics simulations procedure in the Supplemental Materials, Figure S2).

The superposition of the curves for the CD spectra obtained at 10 °C (Figure S3) during heating and cooling shows a discrepancy, which also indicates an irreversible unfolding of the secondary structure of the protein. This unfolding, which can be monitored by circular dichroism spectroscopy, can be used to determine protein stability. However, the thermodynamic analysis of this process is complicated by the irreversible denaturation transition. This may be caused by irreversible protein denaturation or, less likely, by slow kinetics associated with the formation of protein oligomeric forms. Additional studies are required to determine the exact origin of this phenomenon. The objective of the subsequent experiment was to ascertain the presence and size of oligomeric forms in the solution.

### 2.3. Mass Spectrometry Measurements and Gel Electrophoresis

We used an Autoflex Speed time-of-flight mass spectrometer (Bruker Daltonics, Bremen, Germany) to determine the composition of oligomeric forms. MS spectra were recorded in positive ion mode in the 8–190 kDa range using FlexControl software (Bruker Daltonics, Bremen, Germany) and analyzed in FlexAnalysis and Mmass software (http://mmass.org/). The mass spectrum of the ice-binding protein (Table S1) showed a cluster of molecular ions, including protonated ions. The interpretation of the mass spectra was based on the assumption that most recorded peaks are protein molecules of the ice-binding protein and its oligomeric forms.

**Figure 2:**
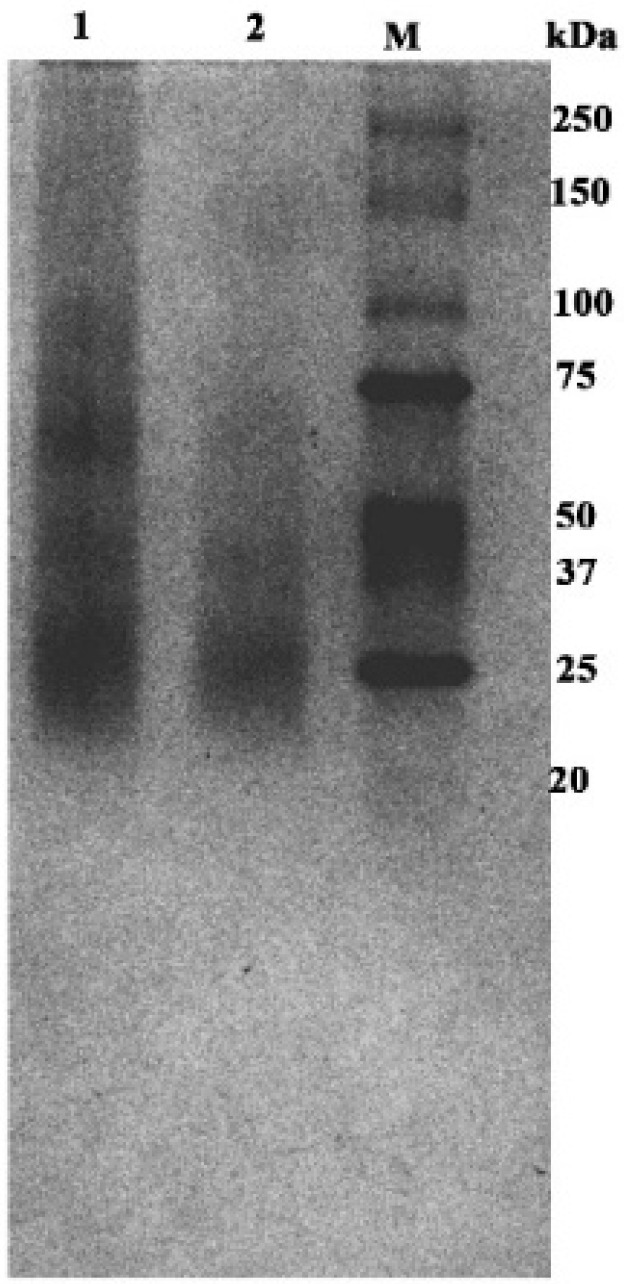
Polyacrylamide 15% gel electrophoresis under native conditions of the oligomeric forms: 1, 2—different samples of protein; M—protein markers of molecular mass (Bio Rad # 1610373).

The masses determined corresponded to the whole ice-binding protein oligomers and not to fragments. The oligomeric forms were characterized by correlating the experimental masses of oligomers with their calculated masses according to the formula *n*∗M, where *n* is the number of monomeric units in the oligomer, and M is the molecular mass of the protein monomer (Table S1). Thus, the mass spectrum of protein detects oligomeric forms corresponding to the dimer (34,828.6 *m*/*z*), trimer (52,245.1 *m*/*z*) and tetramer (69,653.1 *m*/*z*), along with multi-charged ions (M^2+^ and M^3+^, Table S1), allowing us to confirm the mass of oligomeric forms of protein present in solution with higher accuracy. Interestingly, analysis of the mass spectra of the samples obtained after freezing revealed the presence of high-order oligomeric forms of IBP but with low intensity. These forms were pentamer and its doubly charged form (87,145.6 *m*/*z*, 43,573.6 *m*/*z*, respectively, where z = 1 and 2), hexamer (104,609.9 *m*/*z*; 52,245.1 *m*/*z*, where z = 1 and 2) heptamer (122,061.4 *m*/*z* and 61,003.6 *m*/*z*, where z = 1 and 2). The data obtained mass spectrometrically were confirmed by the analysis of the oligomeric forms of the protein by separation by gel electrophoresis in a polyacrylamide gel under native conditions, where oligomeric forms between 25 and 130 kDa were found (Figure 2). Quantitative analysis of the images of native gel bands revealed several ranges of particle distribution based on molecular weight: I (17–30 kDa), II (34–54 kDa), and III (60–75 kDa). These groups are predominantly associated with dimeric, trimeric, and tetrameric forms, respectively. Blurry bands were also present in some samples within the ranges of 80–110 kDa and 120–155 kDa, which indicates the presence of larger oligomeric forms. Spectral images of band intensities are presented in Figure S4.

The character of the gel image shows the large heterogeneity of the sample. Alternative methods were employed to search for oligomeric forms of a higher order, which are difficult to detect using MALDI and gel electrophoresis. In addition, it was essential to ascertain the stability of the complexes (i.e., the thermodynamics of oligomer formation).

### 2.4. AFM Analysis of IBP Oligomerization

**Figure 3:**
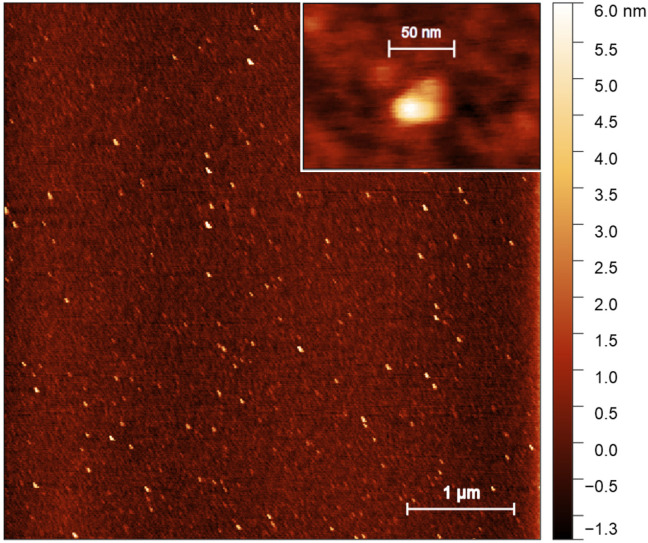
A typical AFM image of the ice-binding protein.

The size of protein was investigated using the AFM method. The characteristic appearance of scanned images of surfaces with deposited proteins is shown in Figure 3,  Figure S5 and  Figure S6. For additional control, mica scans were performed with a protein-free buffer applied to the surface using the same surface preparation protocol as in the case of protein research (Figure S7). No objects with a height of more than 0.5 nm were found on the surface. AFM images were obtained at different protein concentrations in solution (Figure S5). The number of objects in the scanned images decreased at lowering protein concentrations. Taken together, this result indicates that the peaks in the images corresponded to the protein. The data presented for AFM at various protein concentrations reflect rather the limitations of the method and the need for precise selection of conditions for sample preparation. Concurrently, no substantial alterations in the dimensions of protein complexes were observed as a function of protein concentration.

AFM images of the protein contain elongated particles with different sizes. For quantitative image processing, the shape of the particle was approximated by an ellipse. The size of the ellipse semi-major and semi-minor axes, their ratio, and the area and height of the particles were determined as their dimensional characteristics. After analyzing all the images, we obtained a particle size distribution containing several obvious and less pronounced peaks. To carry out a quantitative analysis of the particle size, we identified four ranges on the distribution graph for the semi-major axis size: I (5–20 nm), II (17–27 nm), III (28–44 nm) and IV (44–60 nm) (Table 1). Next, those particles that fall into each range were selected, and the obtained distributions of the particle number from the parameter value (semi-major and semi-minor axis sizes, their ratio, height and surface area) were approximated by the Gaussian function. The particle parameter distributions for all ranges are shown in Figure S6. The average values and standard deviations of the parameters obtained are presented in Table 1.

The average length of the semi-major axis varied from 14.23 nm to 51.06 nm. At the same time, the semi-minor axis size for each region was small, so the semi-axis ratio was in the range of 1.25 to 1.86. The average height of the sample weakly depended on its other dimensions and varied from 1.57 nm to 2.43 nm. Small particles (ranges I and II) were less elongated than larger ones (ranges III and IV). The low particle heights observed in the AFM images (1.57–2.43 nm) can suggest the softness of the protein particles dispersed on the surface. At the same time, it can also speak in favor of forming planar complexes. Thus, AFM analysis data obtained indicate several different size particles (oligomeric forms) of the protein form in solution.

### 2.5. Small-Angle X-Ray Scattering Analysis of IBP Oligomerization

**Figure 4:**
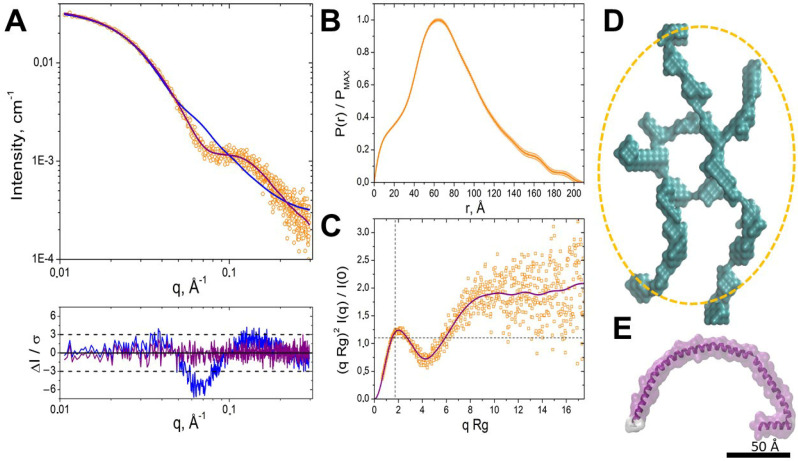
SAXS analysis of the ice-binding protein solution structure. (**A**). Experimental *I*(*q*) profile for IBP (orange circles). The blue curve corresponds to the experimental data fit by the elliptical cylinder model obtained with the SasView programme. The purple curve corresponds to the DAMMIN approximation. From below: normalized residuals plot for elliptical cylinder and DAMMIN ab initio approximations (blue and purple, respectively); (**B**). Normalized pair distance distribution function *P*(*r*); (**C**). Dimensionless Kratky plot. The purple curve corresponds to the regularized *I*(*q*) from the IFT approximation. The dashed horizontal and vertical lines intersect at point (√3, 1.104); (**D**). DAMMIN ab initio structure; The yellow ellipse represents the dimensions of the model achieved using the SasView program elliptical cylinder approximation; (**E**). The AlphaFold2-predicted structure for protein was obtained from the AlphaFold Protein Structure Database (AF-P80961-F1-v4, UniProt ID: P80961). A scale bar of 50 Å applies to panels.

We employed SAXS to analyze the structural features of IBP. Scattering profile I(q) of the protein (Figure 4A), instead of a strictly decreasing dependence, shows an inflexion at 0.07 0.12 Å^−1^, after which decreasing dependence continues. The *P*(*r*) function, shown in Figure 4B, features the main peak at ~65 Å and a secondary, unresolved peak at 10–15 Å. Both of these patterns—an inflexion point or a peak in the *I*(*q*) profile and a secondary peak in the *P*(*r*) function at smaller distances—could be observed for multiphase systems (such as detergent–protein complexes) or systems exhibiting a correlation length between parts of the particles [39]. However, since the protein we are studying is water-soluble and purified without adding detergents, we can consider only the latter scenario as an explanation.

The calculated radius of gyration Rg obtained from the Guinier approximation (Figure S8A) and indirect Fourier transform (IFT) is close to 60 Å (see details in Table S2). The maximum particle diameter Dmax, corresponding to IFT fit in the GNOM programme, is determined as 208 Å. BIFT programme calculation for Dmax resulted in an equal value of 204 Å.

The dimensionless Kratky plot (Figure 4C) provides additional insights into the protein conformational state. It demonstrated a mixed profile, with a bell-shaped peak with a maximum position near the point (√3, 1.104), typical for compact structures, and a plateau at high qRg-values corresponding to flexible or disordered proteins [40].

A comparison of SAXS data with AFM results revealed notable consistency in structural dimensions. AFM analysis identified a class of particles with dimensions matching Dmax, obtained by SAXS data analysis (~20 nm in diameter) and the secondary P(r) correlation peak (~10–20 Å thickness). These particles (see data for the range I in Figure S8) represented the smallest class observed in AFM (Figure S6, range I), suggesting that SAXS samples contained the most compact fraction. There were differences in sample preparation for SAXS and AFM: samples for AFM analysis were prepared in 20 mM HEPES buffer, pH 7.5, containing 100 mM sodium chloride. For small-angle X-ray scattering (SAX) analysis, the samples were prepared in phosphate buffer, pH 6.5, and 100 mM sodium chloride and subjected to more enhanced freezing at −80 °C. Sample IBP freezing at low temperatures might have enriched specific particle populations in the SAXS analysis. As a particle thickness of about 16 Å was observed in AFM, further structural modelling with an elliptical cylinder model was performed, assuming a fixed thickness of 16 Å, and the major and minor axes were optimized. The resulting model had a minor axis of ~77 Å and an axis ratio of ~1.2, suggesting a slight elongation. While the approximation curve provided a reasonable fit in the low-q region (q < 0.04 Å^−1^), the fit deteriorated in the high q-range, with a reduced χ2 of ~4.3 (Figure 4 panels A, blue curves). This result suggested that a more complex structural model was required for a more reliable description of the experimental data.

The ab initio shape reconstruction using the DAMMIN programme provided a more accurate data approximation, with χ2 = 0.93 (Figure 4A, purple curves). This approach resulted in models that appeared as elongated, loosely packed assemblies rather than tightly folded globules (Figure 4D). According to the overall dimensions and configuration of the model, it is comprised of several protein monomers presented by α-helices (see AlphaFold2-predicted structure in Figure 4E). Additional evidence supporting the prominence of α-helical fragments comes from the Guinier plot for elongated particles (ln(qI(q)) vs. q2, Figure S8), which yields Rc = 4.86 ± 0.35 Å. This evidence corresponded to a diameter of 2 Rc √2 = 13.7 ± 1.0 Å (see Table S2), closely matching the ~12 Å diameter of an α-helix. The absence of dense packing suggests inter-monomer contacts with a limited number of specific interaction sites. These observations contradict structural predictions for oligomers, which tend to maximize intermonomer contacts.

The obtained SAXS data approximation reveals a minimum structure dimension of 40 Å, which exceeds the AFM-derived thickness of 10−20 Å. However, the thickness distribution obtained by AFM, corresponding to one or two α-helix diameters, is consistent with the SAXS model of an oligomer composed of elongated α-helical elements. This discrepancy could be attributed to the specific conditions of AFM sample preparation. During immobilization on the substrate, flexible protein regions that normally extend freely in solution might have adhered to the substrate, effectively «flattening» the structure. This projection effect could result in the apparent reduction of particle thickness in AFM measurements corresponding to a range of one to two α-helix diameters, consistent with the observed distribution.

The ab initio model suggests that protein forms oligomers. Molecular weight estimations support the assumption of a tetrameric or pentameric assembly. The excluded volume of the reconstructed SAXS model (~105 nm^3^) corresponds to ~80–85 kDa, while the molecular weight of the monomer was 17.5 kDa, including the His-tag and linker. Furthermore, oligomeric forms, including the tetramer and pentamer, have been corroborated through mass spectrometric analysis (see Tables S1 and S2). Non-denaturing PAGE (polyacrylamide gel electrophoresis) analysis also confirms these findings, revealing a band corresponding to four to five times the molecular weight of the monomer. The broadness of the band is due to the loose quaternary structure of the protein.

To conclude, SAXS revealed that IBP formed elongated assemblies with limited intermonomer interfaces. The SAXS and AFM data combinations indicated a structure that combines compactness with flexibility. The details of the ice-binding protein oligomers observed from these methods underscore the importance of complementary techniques to resolve structural features.

### 2.6. Isothermal Titration Calorimetry Analysis of IBP Oligomerization

**Figure 5:**
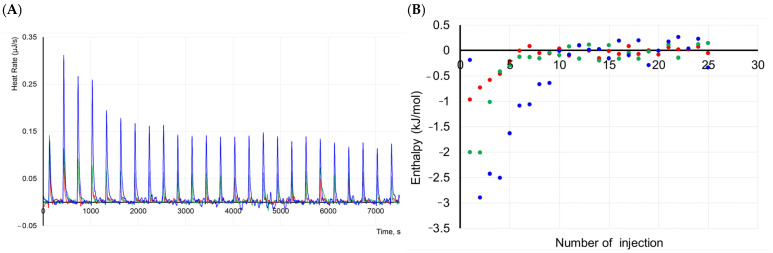
ITC analysis of protein complex dissociation. (**A**). The thermograms and (**B**) binding isotherms obtained by integration of peaks in the thermograms at 15 (green), 25 (blue), and 37 °C (red) at a protein concentration of 0.1 mM titrated in buffer containing 100 mM NaCl, 20 mM HEPES, pH 7.5.

The dissociation of the protein complexes was analyzed using ITC at different temperatures (15 °C, 25 °C and 37 °C). The thermograms and binding isotherms constructed by integrating all peaks in the thermograms are shown in Figure 5.

The data obtained indicate increasing the heat of protein complex dissociation at lowering the temperature. The thermal effect can be associated with a decrease in the number of proteins in oligomers or with high enthalpy values of the formation of the oligomers. MS analysis showed that the formation of oligomeric forms in solutions and native electrophoresis illustrated non-stable complexes. Moreover, the dynamic process of oligomerization occurred in the protein solution.

Complex stabilization at lower temperatures suggests that IBP needs a large surface area to bind more effectively to ice crystals. It can be proposed that the protein can exist in a solution in various oligomeric forms, including high-order oligomers. The formation of high-order oligomers can occur through the combination of monomeric units as well as through the combination of various oligomeric forms. For example, a dimer can form a high-order oligomer with a trimer. Therefore, a quantitative analysis of the data obtained was hindered.

### 2.7. Molecular Modeling of a Protein Complex with Ice

The combination of data obtained by various methods (AFM, SAXS, non-denatured PAGE, mass spectrometry) demonstrates the presence of various oligomeric forms of the protein. Moreover, their analysis suggests that predominantly tetrameric forms are formed in solution. Consequently, the next stage of our research involved acquiring three-dimensional structures to facilitate modelling a promising complex with ice. Utilization of the GalaxyWeb [41] server facilitated acquisition of tetrameric structures (Figure S9).

A comparison of the spatial characteristics of the models obtained as a result of molecular modelling (Table S3) with experimental data determined by AFM methods showed that model No. 4 (Figure S9) best agrees with the results obtained. The model’s dimensions (15.4 nm) correspond to range I (5–20 nm, Table S3), as determined through analysis of AFM data. In the interpretation of the data, the potential for the formation of multilayer structures in the AFM analysis was given due consideration. The selected model is distinguished by the presence of the majority of amino acid residues on the surface, which are hypothesized to be involved in interaction with ice. Furthermore, model No. 4 contains histidine tails at the termini of polypeptide chains that do not contribute to the formation of oligomeric complexes. The obtained structure does not align precisely with the data obtained using the SAXS method. This may be due to the fact that the data were obtained without taking into account the polydispersity of the system.

**Figure 6:**
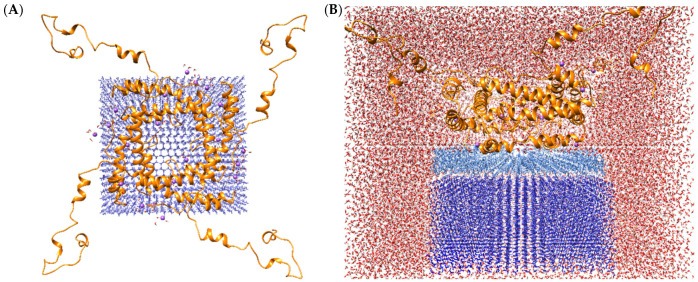
The structure of the complex of tetramer protein (**A**) binding to the surface of ice model 1 h: The protein molecule is colored orange, sodium ions is colored purple, the layer of ice is colored blue. The model illustrates the structural configuration of a tetrameric protein in the presence of ice within an aqueous environment (**B**). The protein molecule is represented by the color orange, the layer of ice is colored blue, while the layer of water between the protein and the ice surface is represented by the color light blue; The red background shows that part of the water remains unfrozen.

Figure 6 provides a visual representation of the tetramer protein complex binding to the ice surface. Molecular dynamics was performed using Amber20 software with accelerated GPU code and ff19SB force fields for protein (as demonstrated to be most effective) and tip4p/ice [42] for ice and tip3p for solvent. The initial system was minimized in an implicit solvent model to relax the protein molecule. The complex was then dissolved in tip4p water and minimized to relax the solvent molecules while holding the protein and ice. The subsequent procedure entailed a gradual heating process, with the target temperature set at 269 K. Molecular dynamics were performed at 269 K for 500 ns. The root mean square deviation (RMSD) for the protein in the explicit solvent reached a maximum of approximately 11 Å. The oligomer structure stabilized at 300 ns, with His6 and the TEV protease cleavage site contributing most to the molecule’s fluctuation. The surface of the ice-binding substance exhibits a high degree of hydrophobicity. Concurrently, hydrophobic groups facilitate a quasi-liquid state of water between the protein surface and the ice crystal. The advantage of this oligomeric form is the ability to interact with several ice planes simultaneously.

## 3. Materials and Methods

### 3.1. Purification of Ice-Binding Protein

The plasmid with an embedded gene encoding ice-binding protein P80961 (UNIPROT, [43]), containing the His_6_ N-terminal label and the TEV protease cleavage site (MENLYFQS), was obtained from Eurogen LLC (Moscow, Russia). The plasmid containing the target gene of the IBP was transformed into chemically competent *Escherichia coli* BL21 cells according to the standard protocol described in Chang et al. [44]. Then, the protein was expressed in *E. coli* BL21 by growing in Luria Broth medium supplemented with Kanamycin (50 µg/mL) (Fluorochem, Hadfield, UK) at 37 °C. When OD_600_ of the culture reached 0.6, protein expression was induced by adding Isopropyl-β-D-1-thiogalactopyranoside (Thermo Fisher Scientific, Abingdon, UK) to a final concentration of 0.5 mM and growing for 5 h at 37 °C (shaking 220 rpm). The cells were lysed using a Sonopuls HD 4100 ultrasonic cell homogenizer (Bandelin Electronic GmbH & Co. KG, Berlin, Germany) by conducting 16 ultrasound treatment cycles of 15 s on ice. The resulting homogenate was centrifuged at 30,000× *g* for 1 h. The clarified lysate was bound to a HisTrap HP column (5 mL; GE Healthcare, Chicago, IL, USA). The recombinant (histidine)6-tagged protein was eluted by a two-step gradient of imidazole (step 10% for deleting non-specific protein and 65% for elution protein) in buffer (20 mM Tris-HCl, pH 8.0, 300 mM NaCl, 500 mM Imidazole), followed by additional purification by HiLoad 26/600 Superdex 75 (Cytiva, GE Healthcare Life Sciences, Chicago, IL, USA). The fractions were pooled and concentrated using a 3 kDa cut-off spin column (Cytiva, GE Healthcare Life Sciences, Chicago, IL, USA) at 4 °C. The concentration of the purified protein was determined using ultraviolet (UV) absorbance at 280 nm on a NanoDrop OneC device (Thermo Scientific, Medison, WI, USA). The extinction coefficient is 2980 (M·cm)^−1^.

The purified protein was characterized on an Orbitrap Q Exactive HF high-resolution mass spectrometer (Thermo Scientific Inc., Waltham, MA, USA) after proteolysis by trypsin in the 200–1600 Da range. The peptides were separated by 2% phase B (0.1% formic acid and 100% acetonitrile) for 3 min at a flow rate of 250 mL/min and then eluted with a gradient B (from 2% to 40%) over 8 min and a gradient B (from 40% to 95%) over 25 min on column Poroshell 120 EC-C18 (Agilent Tech, Waldbronn, Germany). The spray voltage was set at 4.2 kV, and the normalized collision energy was set at 30% for MS/MS. Data-dependent ion selection was performed using the most abundant 10 ions from a full MS scan for MS/MS analysis.

Identification of protein was conducted using the Proteome Discoverer programme (version 3.1, Thermo Fischer Scientific, Waltham, MA, USA), using the SEQUEST algorithm. The search was completed in a local database containing an amino acid sequence of protein with a His-tag. The amino acid sequence of IBP was obtained from the Uniprot database (P80961, https://www.uniprot.org/ (accessed on on 6 December 2023)). The following settings were applied: an MS error tolerance of 10 ppm, an MS/MS error tolerance of 0.02 Da, trypsin as the protease, oxidation (M) as a variable modification, with a peptide confidence level of low (Figure S1).

### 3.2. Circular Dichroism Spectroscopy

Circular Dichroism (CD) spectra were recorded using a J-600 spectropolarimeter (JASCO, Tokyo, Japan) in a 10 mm path length cell in the 200−260 nm wavelength range at 1 nm resolution. Each measurement was repeated 12 times, after which the spectra were averaged. The recombinant protein solution was at a concentration of 0.2 mg/mL in buffer 20 мM HEPES (Molekula GmbH, München, Germany) pH 7.6, 100 мM NaCl (Sisco Research Laboratories SRL, Mumbai, India). In experiments to assess thermal stability, spectra were collected by measuring the CD signal at different temperatures ranging from 0.2 °C to 80 °C. The cuvette temperature was maintained using a circulating water bath (Huber Kiss K6, Offenburg, Germany). The CD spectra, which were obtained through experimental means and then averaged for each temperature, were loaded and processed using the Bestsel online resource (https://bestsel.elte.hu/index.php (accessed on 1 December 2025)). This operates on the basis of the DSSP algorithm [45]. The algorithm in question is designed to calculate the most probable secondary structure, with the consideration of the tertiary structure of the protein. The formation of the protein folding type is initiated by conducting a search among PDB structures that exhibit a comparable secondary structure composition, as determined by their Euclidean distance. Subsequently, the folding classification of these structures is determined.

Experimental denaturation/renaturation curves (Figure 1B) were constructed based on the values obtained at a wavelength of 222 nm, since one of the peaks that is characteristic of α-helical proteins is observed at this wavelength.

The graphs (Figure 1C) demonstrate the contribution of each element to the secondary structure of the protein and its change upon heating.

These were analyzed using nonlinear approximation via the least squares method.

The curves in Figure 1B,C are constructed only for clarity of the presented data.

### 3.3. MALDI Mass Spectrometry

The protein at a concentration of 0.4 mM was pre-desalted using a pipette tip (volume is 10 μL) ZipTip (Millipore, Bedford, MA, USA) containing C4 reversed-phase media by standard protocol. Samples bonded with media of ZipTip pipette tip previously equilibrated 0.1% trifluoroacetic acid (TFA) (Sigma-Aldrich, St. Louis, MO, USA) in water. The sample was washed 10 times with 0.1% TFA solution.

The matrix was prepared according to the protocol described in Signor et al. [46]. The protein sample was deposited by aspirating 1 μL of elution solution [acetonitrile (ACN) (Biosolve, Dieuze, France) in 0.1% TFA (70:30, *v*/*v*)] into the ZipTip pipette tip on the previously prepared α-cyano-4-hydroxycinnamic acid (α-CHCA) (Sigma-Aldrich, St. Louis, MO, USA) thin layer. Immediately after that, 1 μL of the matrix solution 20 mg/mL α-CHCA solution in ACN and 5% formic acid (Sigma-Aldrich, St. Louis, MO, USA) (70:30, *v*/*v*) was added. The sample and the matrix were mixed on the target MTP 384 ground steel target (Bruker Daltonics, Bremen, Germany) and allowed to dry at ambient temperature.

The mass spectra were recorded on an Autoflex Speed mass spectrometer MALDI (Bruker Daltonics, Bremen, Germany) equipped with a nitrogen laser (337 nm) (VSL-337 ND, Laser Science, Newton, MA, USA) with a pulse duration of 1−5 ns in the positive linear mode in the range 8–190 kDa using the FlexControl Version 3.3 software (Bruker Daltonics, Bremen, Germany, last updated on 1 December 2023). They were subsequently analysed in FlexAnalysis Version 3.3 and Mmass software Version 5.5.0 (mmass.org, last updated on 1 December 2023). A standard MTP target prepared according to specifications provided by Bruker Daltonics (Bremen, Germany) was used for external calibration.

### 3.4. Native Electrophoresis

Oligomeric forms of protein in sample buffer (250 мM Tris, pH 6.8; 12.5% glycerol; 0.025% Bromophenol blue) were analysed using 15% polyacrylamide gel (acrylamide/bis-acrylamide solution 32:1, 40%T, 3%C) electrophoresis in buffer (1.5 M Tris-HCl pH 8.8) in a WIX-miniPRO (WIX Technology Beijing Co., Ltd., Beijing, China). Electrophoresis was conducted for 1–2 h at room temperature in the buffer: 25 mM Tris-glycine, pH 8.3 at 100–170 V with subsequent Coomassie Brilliant Blue staining followed by gel scanning on a VersaDoc (BioRad, Hercules, CA, USA). The images were processed in Gel-Pro Analyzer v.4 (Media Cybernetics, L.P., Rockville, MD, USA).

### 3.5. AFM Imaging

AFM images were captured in tapping mode in the air on a Multimode 8 atomic force microscope (Bruker, Bremen, Germany) in «ScanAsyst in Air» mode using ScanAsyst-Air probes with a typical tip curvature radius of 2 nm (Bruker, Bremen, Germany) or in tapping mode with a diamond-like carbon NSG10 series cantilever (NT-MDT, Zelenograd, Russia) with a tip curvature radius of 1–3 nm. The protein sample was diluted in buffer solution (20 mM HEPES pH 7.6, 150 mM NaCl) in 15 mM MgCl_2_ in concentrations of 10^−6^ M, 2 × 10^−6^ M or 4 × 10^−6^ M and 10 μL of the sample was deposited on the ice-cooled surface of mica (0.5 × 0.5 cm) for 1 min. The surface was then rinsed with 3000 μL MilliQ water and dried under a gentle stream of argon. AFM images were processed and prepared using Gwyddion software (version 2.66) [47].

### 3.6. Small-Angle X-Ray Scattering

Preliminary test measurements were performed using the SAXS instrument Rigaku MicroMax-007HF (Rigaku Oxford Diffraction, Woodlands, TX, USA) previously used and described in Tsoraev et al. and Ryzhykau et al. [48,49]. Scattering data presented in the manuscript were collected at the beamline BL19U2 of the National Facility for Protein Science Shanghai at the Shanghai Synchrotron Radiation Facility (SSRF, Shanghai, China) described in Liu et al. and Li et al. [50,51]. The wavelength (λ) of X-ray radiation was set at 1.03 Å. The sample-to-detector distance was set at 2.67 m (corresponding range of momentum transfer q = 4π sin(θ)/λ was 0.006–0.456 Å^−1^, where 2θ is the scattering angle). Scattered X-ray intensities were collected using a Pilatus3 2 M detector (DECTRIS Ltd., Baden, Switzerland) with an exposure time of 1 s and 20 consecutive exposures for the same measured sample.

BioXTAS RAW (version 2) [52] and ATSAS (version 3.0) [53] software suites were used for data treatment. Programmes GNOM from ATSAS [54] and BIFT (Bayesian indirect Fourier transform) from RAW [55] were used for the calculation of pair distance distribution functions P(r) by the indirect Fourier transform method. The DAMMIN [56] programme of the ATSAS online service [57] was used for ab initio bead modelling based on SAXS data.

We used the SasView programme [58] for SAXS data approximation with simple-shaped models. Detailed information about the experimental setup and data analysis are presented in Table S2.

### 3.7. Isothermal Titration Calorimetry

Protein complex dissociation was analyzed using the Nano ITC isothermal titration calorimeter (TA Instruments, New Castle, DE, USA). All ITC measurements were conducted with 200 rpm stirring and a 300 s delay between injections of 2 μL of the sample using a syringe of 50 μL and a sample cell of 190 μL in volume. Different temperatures (15 °C, 25 °C and 37 °C) were used for the ITC analysis. The experiments were performed in a buffer containing 100 mM NaCl, 20 mM HEPES and pH 7.5. The sample concentration was taken at 0.1 mM. The data were processed with NanoAnalyze software (version 3.12.5, TA Instruments, New Castle, DE, USA).

### 3.8. Molecular Dynamics Simulation

Protein structure modeling was performed with His_6_ and a TEV protease cleavage site to obtain results comparable to those obtained in experiments. The tetramer complex with ice was obtained using Chimera 1.16.1. The surface structure of ice Ih was generated using GenIce2 [59] with a size of 76 × 71 × 32 Å to facilitate molecular dynamics simulation and the tip4p/ice model. The Amber20 software [60] was employed for simulations, incorporating the ff19SB force field for proteins [61] and tip3p for the solvent [62]. The simulation was performed at a temperature of 269 K for 500 ns. The initial system was minimized in an implicit solvent model to relax the protein molecule. The complex was then dissolved in tip4p water and minimized to relax the solvent molecules while holding the protein and ice. The obtained trajectories were analyzed using the CPPTRAJ module from AmberTools and Chimera 1.16.1 software [63,64].

## 4. Conclusions

IBPs are promising targets for industrial applications, but the lack of sufficient information on their mechanisms of action is a limitation. We have shown that the formation of different oligomers was supported by the shape and parameters of the protein obtained by AFM and the SAXS results. Establishing the exact composition of the oligomers proved to be a challenging problem, which was not solved unambiguously with the methods used. Nevertheless, we hypothesize that protein forms could be tetrameric or pentameric, formed from monomer units or a combination of these, such as a dimer with a trimer or a tetramer with a monomer. The thermodynamic equilibrium between these multimers was confirmed using ITC analysis. We constructed a tetrameric protein complex with ice and performed molecular modeling, which demonstrated the stability of the resulting complex.

Knowledge of the three-dimensional structure may provide insight into its biological function, molecular mechanisms and interaction sites. A better understanding of the molecular basis of IBP properties is necessary to advance our understanding of the evolution of this highly distinctive cold adaptation strategy and the biological function of IBP. Our results suggest that a molecule containing multiple domains in an oligomeric complex acquires an increased affinity for ice binding. Ice-binding efficiency appears to be enhanced as a result of the increased interaction area between the protein and the ice, which in turn determines the presence of oligomeric forms of IBP. We hypothesize that different oligomeric forms of the protein may form thermodynamically more favorable complexes than monomeric forms, depending on ionic strength or temperature.

## Figures and Tables

**Table 1:** The values of the protein complexes parameters were obtained using AFM analysis. a1—the semi-major axis length, a2—the semi-minor axis length, a1/a2—the semi-axes ratio, $\sqrt{S}$—the square root of the area, z—the height.

Ranges of Values in the AFM Method	a1, nm	a2, nm	a1/a2	$\sqrt{S}$, nm	z, nm	Ranges for the Semi-Major Axis of Size, nm
I	14.2 ± 3.9	10.5 ± 2.8	1.4 ± 0.2	20.1 ± 5.5	1.57 ± 0.31	5–20
II	24.0 ± 2.5	18.6 ± 1.5	1.3 ± 0.1	36.3 ± 3.0	1.86 ± 0.46	17–27
III	32.8 ± 4.6	20.5 ± 2.7	1.5 ± 0.2	44.3 ± 4.2	2.43 ± 0.74	28–44
IV	51.1 ± 4.9	27.5 ± 7.8	1.86 ± 0.5	60.5 ± 10.1	1.90 ± 0.47	44–60

## Data Availability

The original contributions presented in this study are included in the article/Supplementary Material. Further inquiries can be directed to the corresponding author.

## Supplementary Materials

The following supporting information can be downloaded at: https://www.mdpi.com/article/10.3390/ijms262411790/s1.
