# Supplementary material for: The Study of Ice-Binding Protein Oligomeric Complexes
Source: Int J Mol Sci. 2025 Dec 5;26(24):11790. doi: 10.3390/ijms262411790 (PMC12732928; doi:10.3390/ijms262411790)
Supplement: Supplementary file 1 [file ijms-26-11790-s001.zip › ijms-4010248-supplementary.pdf]

Supplementary Materials

The Study of ice-binding protein oligomeric complexes

Galina A. Oleinik[1], Maria A. Kanarskaya[1,2], Na Li[3], Alexander A. Lomzov[1,2], Vladimir V. Koval[1,2], Svetlana V. Baranova[1*]

[1]Institute of Chemical Biology and Fundamental Medicine, Siberian Branch of the Russian Academy of Sciences, Novosibirsk, 630090, Russia
[2]Department of Natural Sciences, Novosibirsk State University, Novosibirsk, 630090, Russia
[3]National Facility for Protein Science Shanghai, Shanghai Advanced Research Institute, Shanghai, 201210, People's Republic of China
**Correspondence:* swb@1bio.ru

A

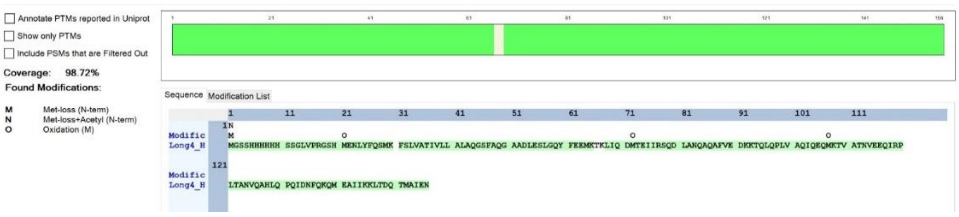

B

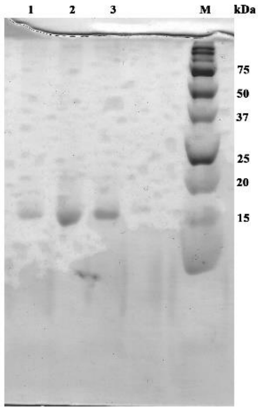

Figure S1. The protein characterisation: A. Peptide coverage map of protein after trypsin digestion; B. Polyacrylamide 18% gel electrophoresis: 1, 2, 3 – Protein samples in various dilutions: 4, 0 (undiluted), and 2 times. The initial concentration was 0.1 mg/ml. M – protein markers of molecular mass (Bio Rad # 1610373)

A

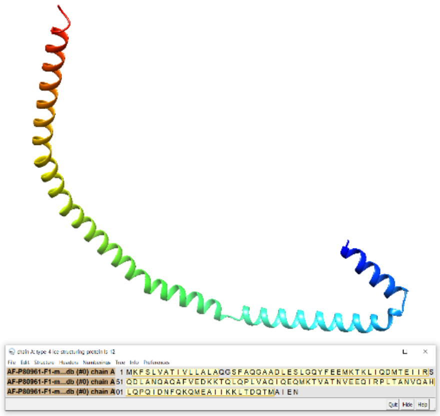

B

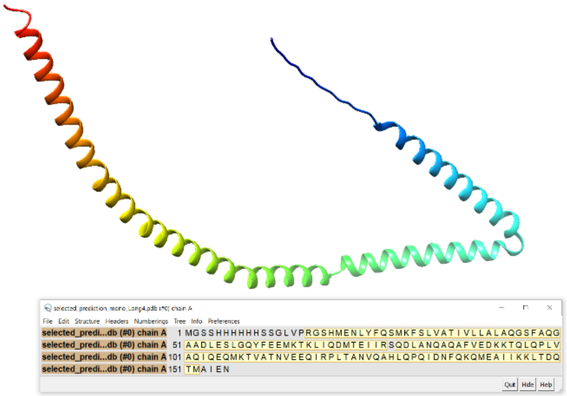

Figure S2. Structure of ice-binding protein from the Longhorn sculpin: A. native protein; B. the protein with a His-tag, linker, and TEV protease cleavage site

Protein molecular dynamics simulations procedure

The ice-binding protein, also known as protein from the Longhorn sculpin (LS-12), currently does not have a structure in the Protein Data Bank (PDB) database. However, using homology modeling with the AlphaFold web server (Jumper et al., 2021), we obtained the structures of two proteins: the protein with a His-tag, linker, and TEV protease cleavage site, and the native protein (fig. S2). Both structures are composed of alpha helices and are in the form of "threads", with a certain distance between amino acid residues of about 16.5 Å, which is consistent with literature data (Oude Vrielink et al., 2016).

Both structures were used for molecular dynamics simulations in an implicit solvent model. Molecular dynamics simulations were performed using Amber20 software (Case et al., 2020), with accelerated GPU code (Salomon-Ferrer et al., 2013; Götz et al., 2012), and ff14SB force fields. The protein fluctuated throughout the 100 ns of the molecular dynamics simulation. Both structures remained stable during the MD trajectory.

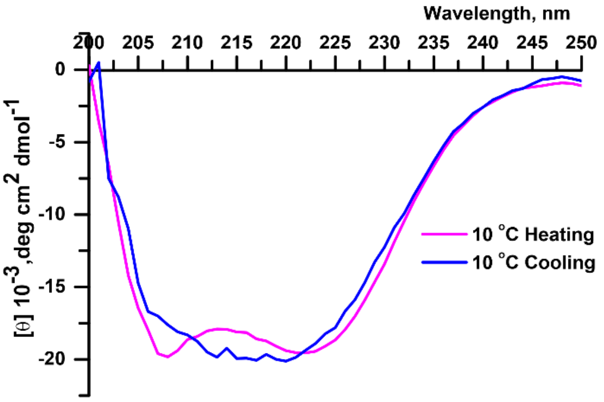

Figure S3. Circular dichroism spectra of protein P80961 from *Myoxocephalus octodecemspinosus* at 10° C. The protein concentration was 0.2 mg/ml, and the buffer was 20 mM HEPES pH 7.6, 100 mM NaCl

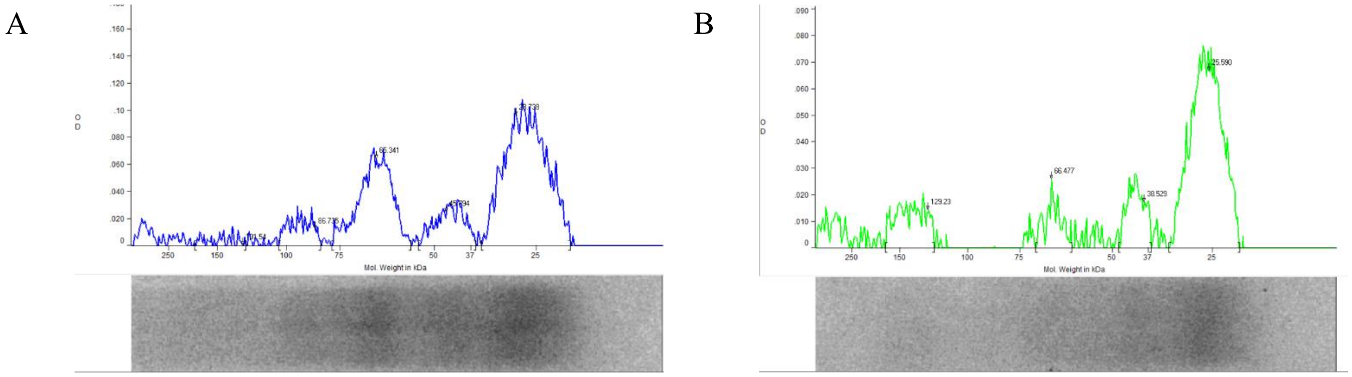

Figure S4. The spectral views of band intensities in gel electrophoresis under native conditions. The spectral views of band intensities in gel electrophoresis under native conditions. corresponding to lines 1 and 2 in figure 2. A. Line 1; B. Lines 2

Table S1. Correlating the experimental masses of oligomers with their calculated masses.

N monomeric units				experimental			MS-spectra
	nM	m/2		nM	m/2	m/3	
1	17418	8709		17418.47	8704.19		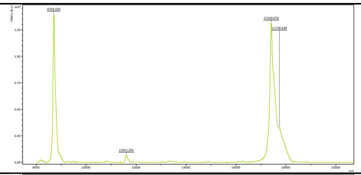
2	34836	17418		34828.69	17418.47	11611.23	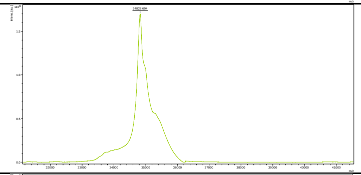
3	52254	26127		52245.17	26124.12	17418.47	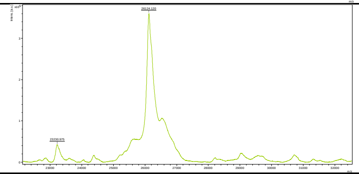
4	69672	34836		69682.48	34828.69	23230.9	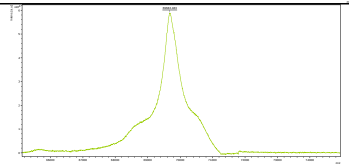
5	87090	43545		87145.63	43573.65		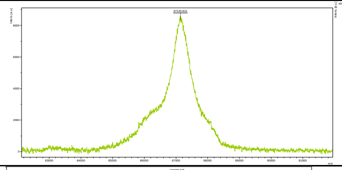 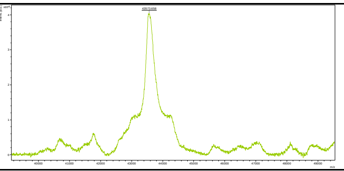
6	104508	52254		104609.97	52245.17		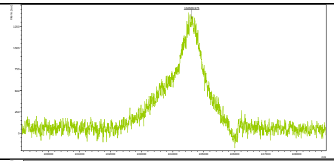
7	121926	60963		122061.48	61003.60		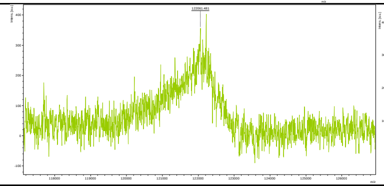 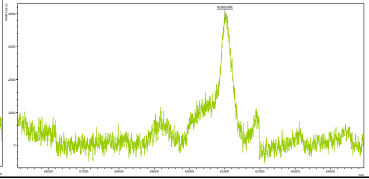

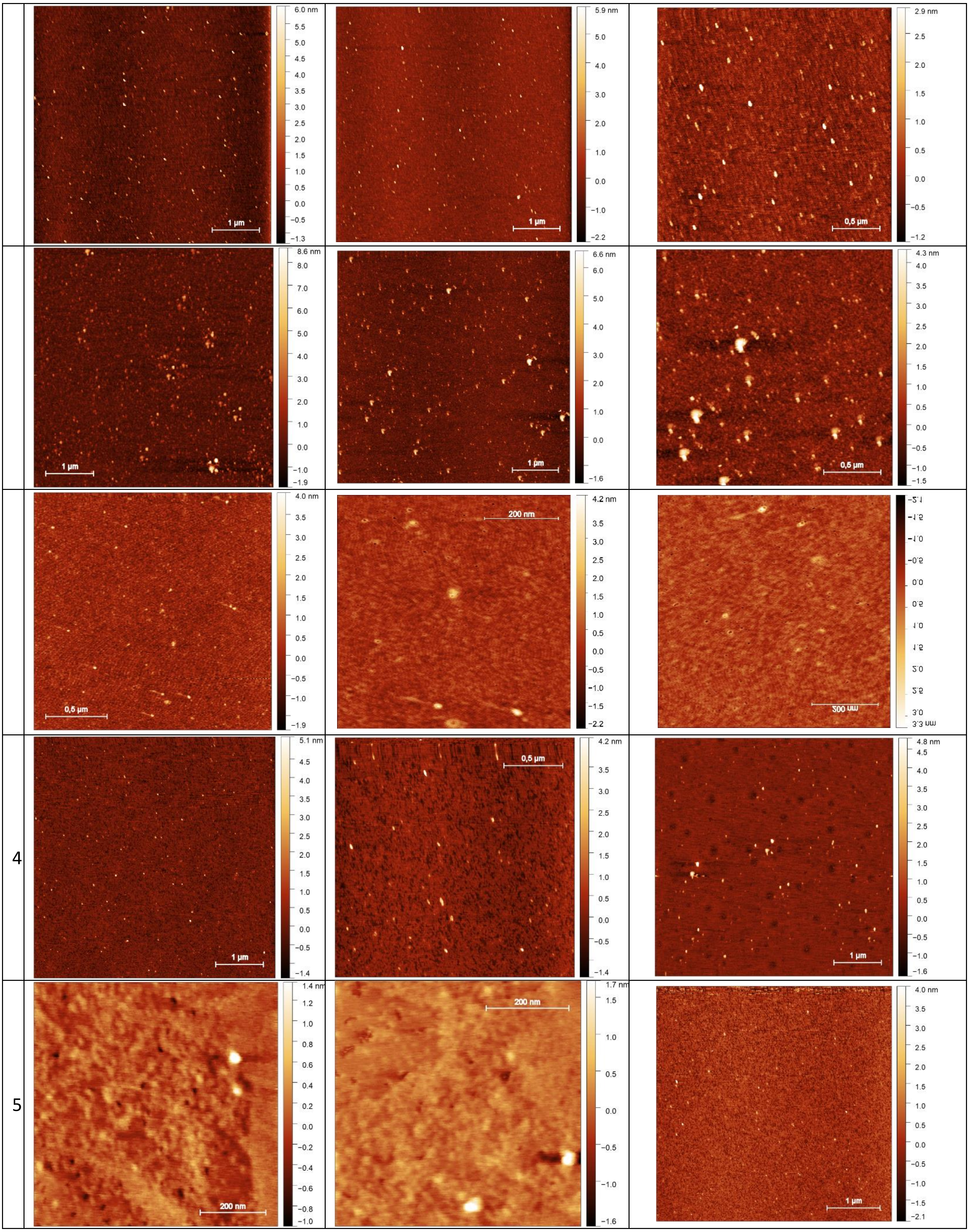

Figure S5. The typical AFM images of Long4 protein. Lines 1-2 correspond to the sample concentration 4×10^{-6} M, lines 3 - 2×10^{-6} M, lines 4-5 - 10^{-6} M

Range	a1, nm (orange, left axis), a2, nm (blue, right axis)	a1/a2	$\sqrt{S}$, m	z, nm
I	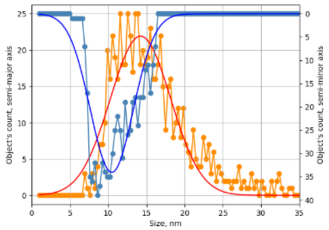	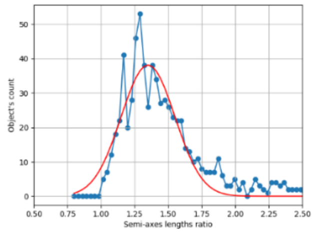	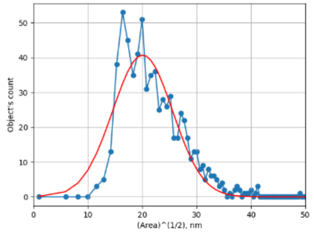	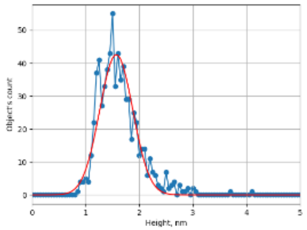
II	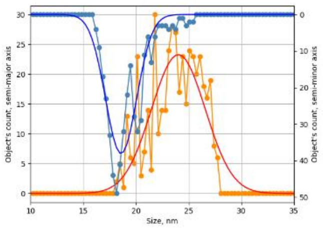	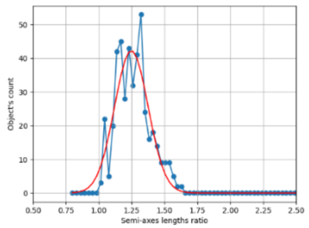	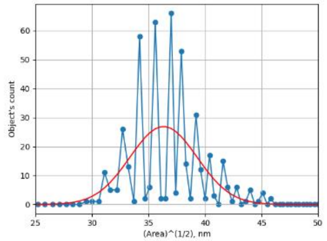	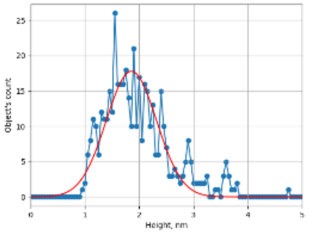
III	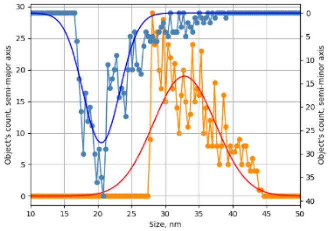	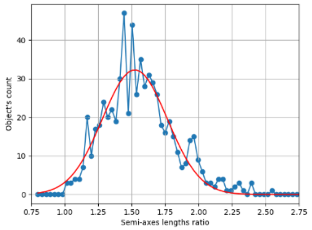	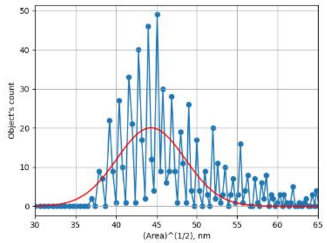	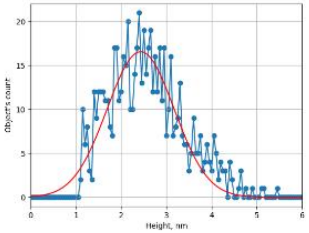
IV	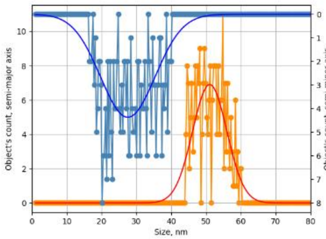	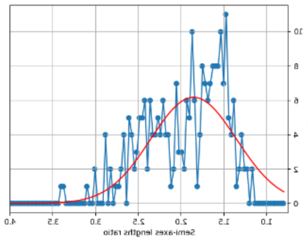	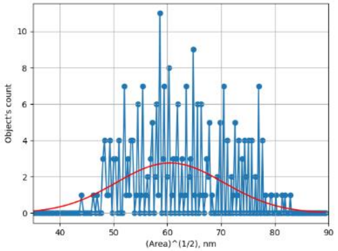	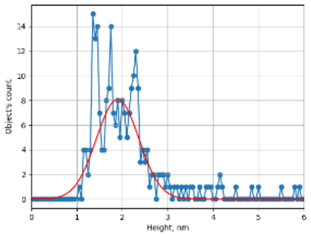

Figure S6. Distributions of protein complexes geometry parameters obtained experimentally using AFM method (dots) and calculated by Gaussian distribution fitting. a1 is the semi-major axis length, a2 - the semi-minor axis length, a1/a2 - the semi-axes ratio, √S - the square root of the area, z – the height

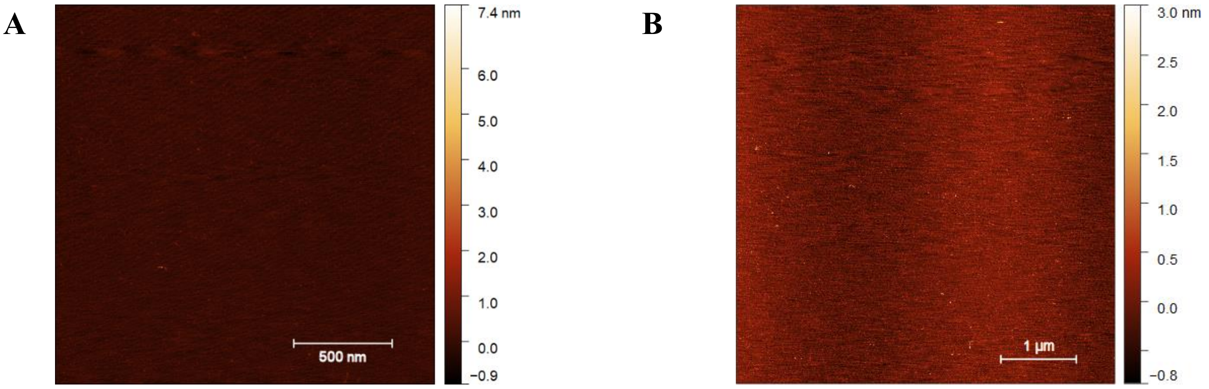

Figure S7. The AFM image: A. the solution for mica (15 mM $MgCl_2$); B. the solution for mica in protein-free buffer

Table S2. SAXS experimental details and data evaluation summary.

***(a)* Sample details**	
Description of sequence	Type-4 ice-structuring protein LS-12<br>Type-4 ice-structuring protein LS-12 from *Myoxocephalus octodecemspinosus* (UniProt ID P80961) with an N-terminal His_6-tag followed by a TEV-protease site
Chemical formula	
Molecular mass (kDa)	17554.00
Approximate volume (Å^3)	
Partial specific volume ν (cm^3 g^{-1})	0.7286
Mean solute and solvent SLD (10^{-6} Å^{-2})	9.458; 12.498
Mean scattering contrast Δρ (10^{-6} Å^{-2})	3.04
Sample concentration (mg ml^{-1})	
Solvent composition	15 mM NaPi, pH 6.4, 67 mM NaCl
***(b)* SAS data collection parameters**	
Instrument	BL19U2 Beamline with Pilatus3 2 M (DECTRIS Ltd) (SSRF, Shanghai, China)
Wavelength (Å)	1.033
Beam geometry (FWHM diameter, sample-to-detector distance)	0.33 mm (H) × 0.05 mm (V);<br>sample-to-detector distance = 2.696 m
Sample configuration	Automated sample changer unit with quartz capillary with 10 µm-thick walls and a 1.5 mm path length
q-measurement range (Å^{-1})	0.006 – 0.456
q-scaling method	Calibration standard: silver behenate powder
Basis for normalization to constant counts	To transmitted intensity by direct beam counter
Absolute intensity scaling method	Comparison with scattering from pure H_2O
Exposure time	7 sec
Sample temperature (°C)	20
***(c)* Software employed for SAS data reduction, analysis and interpretation**	
1D-integration, data averaging and subtraction	RAW 2.2.1
Guinier analysis	ATSAS 2.8.4
Calculation ν values from chemical composition	ProtParam: https://web.expasy.org/protparam/<br>Peptide Property Calculator:<br>http://biotools.nubic.northwestern.edu/proteincalc.html
Calculation of scattering length density (SLD) values from chemical composition	SLD calculator:<br>http://www.ncnr.nist.gov/resources/activation/
P(*r*) calculation	GNOM from ATSAS 4.0.0
Modelling by simple-shaped bodies	SasView 4.2.2

Shape/bead modelling	DAMMIN (ATSAS online)
Atomic structure modelling and fitting the data	CRYSOL from ATSAS 4.0.0
Molecular graphics	VMD 1.9.3
**(*d*) Structural parameters**	
Guinier analysis for globular particles	
$I(0)$ (cm^{-1})	$(3.56 \pm 0.04)\times10^{-2}$
R_g (Å)	58.2 ± 1.3
q-range (Å^{-1})	0.01064 – 0.02264
qR_g - range	0.6191 – 1.3171
Guinier analysis for elongated (rod-like) particles	
$I(0)$ (a.u.)	$(156.6 \pm 9.7)\times10^{-6}$
R_c (Å)	4.86 ± 0.39
Characteristic diameter ($2R_c\sqrt{2}$) (Å)	13.7 ± 1.1
q-range (Å^{-1})	0.1511 – 0.2185
qR_c - range	0.732 – 1.06
$P(r)$ analysis	
$I(0)$ (a.u.)	$(3.66 \pm 0.03)\times10^{-2}$
R_g (Å)	58.2 ± 1.3
D_{max} (Å)	210
q-range (Å^{-1})	0.0106 – 0.2962
$q_{min}\, d_{max} / \pi$	0.7086
Regularization parameter α (a.u.)	4.611
Total quality estimate (GNOM)	0.6993
Experimental MW (kDa)	86.6<br>(from excluded volume of DAMMIN model, see *(f)*)
***(e)* Approximation with simple-shaped models**	
Method	SasView 4.2.2<br>The model of elliptical cylinder.<br>Category: Cylinder; model: elliptical_cylinder.
q-range for fitting (Å^{-1})	0.0106 – 0.2962
Calculated parameters	L = 16 Å (the value was fixed in accordance with AFM results; see main text for the details)<br>R = (76.9 ± 1.9) Å<br>**ν** = 1.18 ± 0.06 (**ν** R = 90.7 Å)
Reduced χ^2	4.3354
**(*f*) Shape modelling results**	
Method	DAMMIN<br>The cylinder with radius of 100 Å and height of 40 Å was used as a search volume.
q-range for fitting	0.0106 – 0.2962
S	P1 / Unknown
Reduced χ^2	0.9272
Total excluded DAM volume (Å^3)	104789
Model resolution (as $2\pi/q_{max}$) (Å)	

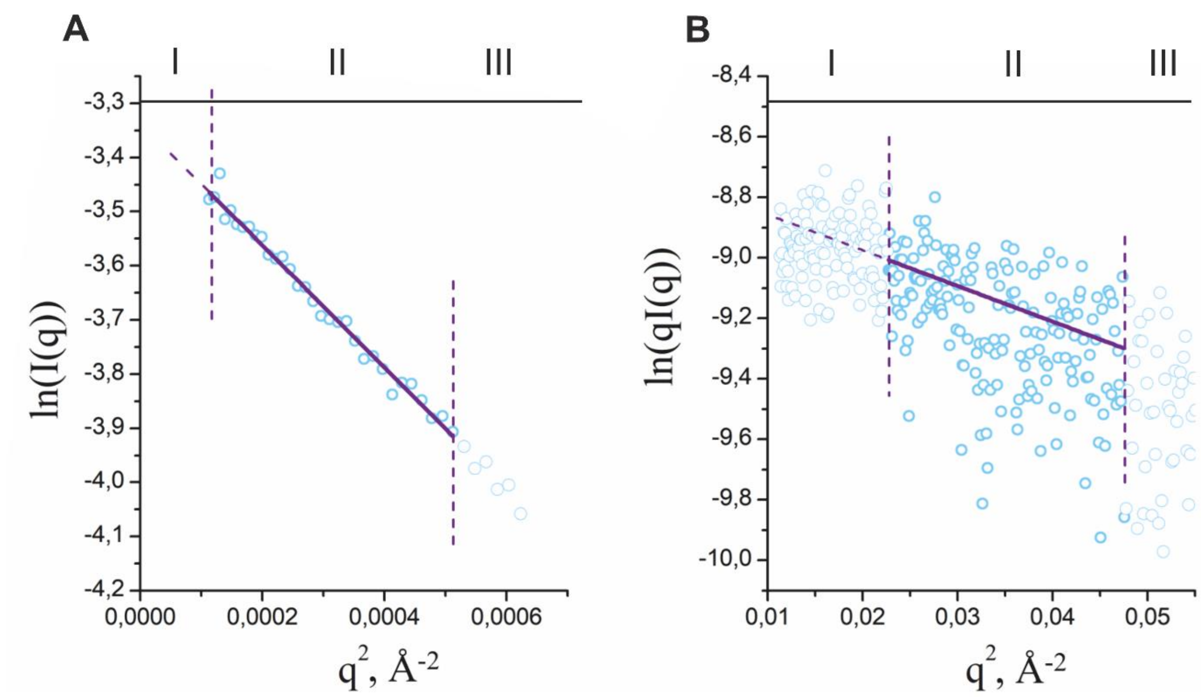

Figure S8. Guinier approximations for SAXS data of protein from Longhorn sculpin. A. Guinier plot for globular particles; B. Guinier plot for elongated (rod-like) particles

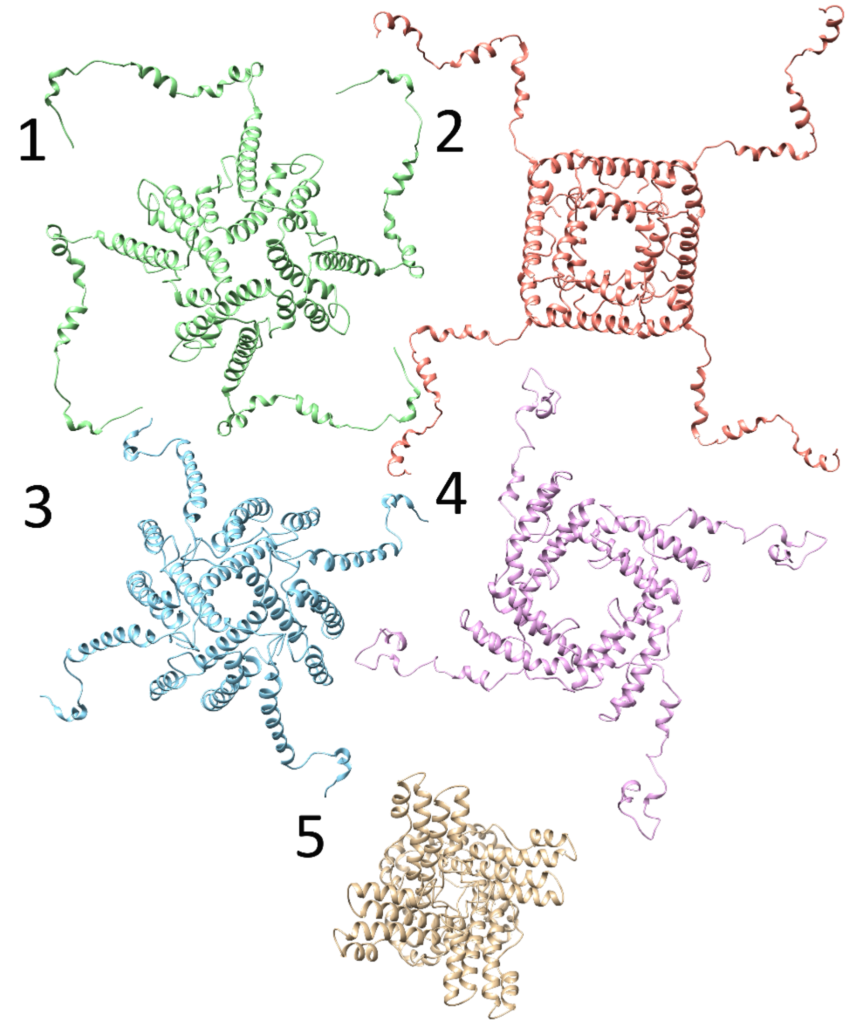

Figure S9. Theoretical structures of IBP tetramers obtained using GalaxyWeb

Table S3. The values of the parameters of the tetramer models.

Particle size groups corresponding to the models	Size of particles in the AFM, nm	№	Size of model, nm	High of model, nm
I	5 – 20	1	14.9	9.9
I, II	5 – 20, 17 – 27	2	18.8	6.8
I	5 – 20	3	12.8	8.0
I	5 – 20	4	15.4	3.3
I	5 – 20	5	7.9	7.5
